# Insights Into Medication Adherence Among Patients With Chronic Diseases in Jeddah, Saudi Arabia: A Cross-Sectional Study

**DOI:** 10.7759/cureus.37592

**Published:** 2023-04-14

**Authors:** Mohammed S Fallatah, Ghassan S Alghamdi, Abdulaziz A Alzahrani, Mohannad M Sadagah, Turki M Alkharji

**Affiliations:** 1 General Practice, King Abdulaziz University, Jeddah, SAU; 2 General Practice, University of Jeddah, Jeddah, SAU; 3 Family Medicine, Al-Thaghr Hospital, Jeddah, SAU

**Keywords:** socio-economic factors, saudi arabia, cross-sectional study, chronic diseases, medication adherence

## Abstract

Background

Non-adherence to medication is a common problem in managing chronic diseases, which are a significant public health concern globally. This study aimed to identify factors related to medication adherence among patients with chronic diseases in Saudi Arabia.

Methods

A cross-sectional survey design was used to collect data through an online survey administered to 400 patients with chronic diseases residing in Jeddah between January and March 2023. The survey included questions about socio-demographic characteristics, chronic disease diagnosis, medication adherence, and factors that may influence medication adherence.

Results

This study recruited 400 participants and found that the majority were female, with a mean age of 46.2 years, and most had at least one chronic disease, with hypertension and diabetes being the most common. The medication adherence score for the entire sample was 5.4, indicating moderate adherence. Overall, 22.9% of study participants had poor adherence to medications. Factors associated with medication adherence included age, gender, and education level, with older age, female gender, and higher education being positively associated with adherence. Medication-related factors such as the number of medications prescribed, medication complexity, and medication cost were also found to be significantly associated with medication adherence.

Conclusion

Our study of medication adherence among chronic disease patients in Saudi Arabia found that adherence rates were moderate, with several factors significantly associated with better adherence. Specifically, older age, female gender, and higher education level were positively associated with better adherence, while a higher number of prescribed medications, more complex medication regimens, and higher medication costs were all significant predictors of poorer adherence.

## Introduction

Chronic diseases are long-term health conditions that are often associated with high morbidity, mortality, and healthcare costs. These diseases include conditions such as hypertension, diabetes, and cardiovascular disease, among others. Chronic diseases are prevalent worldwide, and their prevalence is increasing [1]. In Saudi Arabia, chronic diseases are a major public health concern, with high prevalence rates reported for diseases such as diabetes, hypertension, and obesity [2].

Medication adherence is an essential aspect of chronic disease management and is critical to achieving positive health outcomes. Medication non-adherence is a common problem, with estimates suggesting that up to 50% of patients with chronic diseases do not adhere to their medication regimens [3]. For example, a systematic review found that among pre-dialysis chronic kidney disease (CKD) patients, medication adherence was affected by factors related to the patient's condition, therapy, health system, and socio-economic domains, with an overall pooled adherence rate of 67.4% [4]. Non-adherence can lead to suboptimal treatment outcomes, increased morbidity and mortality, and increased healthcare costs [5]. Therefore, understanding the factors that influence medication adherence is crucial to developing effective strategies to improve adherence and ultimately improve health outcomes.

Despite the high burden of chronic diseases in Saudi Arabia, limited research has been conducted on medication adherence among affected patients. A cross-sectional study conducted in Tabuk found that 76.44% of chronic disease patients were adherent to their medications, with side effects and forgetfulness being the primary reasons for nonadherence [6]. Several factors have been identified as determinants of medication adherence, including patient-related factors, healthcare provider-related factors, and medication-related factors. Patient-related factors include socio-demographic characteristics, psychological factors, and health beliefs [7]. Healthcare provider-related factors include communication, trust, and support from healthcare providers. Medication-related factors include the complexity of the medication regimen, side effects, and cost.

Given the complexity of medication adherence and its multiple determinants, a comprehensive approach is needed to develop effective interventions to improve adherence. Therefore, this study aimed to identify the factors related to medication adherence among patients with chronic diseases in Jeddah, Saudi Arabia.

## Materials and methods

Study design

The study was a cross-sectional survey design. Data was collected through an online survey administered to patients with chronic diseases residing in Jeddah, Saudi Arabia. The study aimed at identifying factors related to medication adherence among patients with chronic diseases in Jeddah, Saudi Arabia. The study was conducted between January and March 2023.

Sampling strategy

The sample for this study was selected using convenience sampling. An online survey link was shared through social media platforms (e.g., Twitter, Facebook, and WhatsApp), and participants were asked to forward the survey to their friends and family members who met the inclusion criteria.

Sample size calculation

A sample size calculation analysis was conducted to estimate the required sample size using a power analysis based on a medium effect size of 0.5, a significance level of 0.05, and a power of 0.80. The analysis indicated that a minimum of 385 participants would be required to detect a significant effect. To ensure adequate power and a sufficient sample size to detect any potential subgroup differences or account for any potential attrition, at least 400 participants were included in the study. A post-hoc power analysis using the effect size obtained from the study and the sample size of 400 yielded a power of 0.89, indicating that the sample size was adequate for the analyses conducted in this study.

Inclusion criteria

Participants were included in this study if they met the following criteria: (1) were adult patients (18 years or older) diagnosed with at least one chronic disease, which is defined as a long-term medical condition that persists for a year or more and requires ongoing medical attention or limits daily activities, (2) had been prescribed at least one medication for their chronic disease by a primary care clinic specializing in chronic diseases, (3) lived in Jeddah, Saudi Arabia, and (4) could read and write in Arabic or English.

Data collection

The data collection tool for this study was an online survey developed by the research team. The survey was available in both Arabic and English and included questions about participants' socio-demographic characteristics, chronic disease diagnosis, and medication-related factors such as the number and cost of medications. The survey was hosted on Google Forms, and the link was shared with potential participants through various social media platforms.

The Medication Adherence Score was assessed using an eight-item questionnaire based on the Morisky Medication Adherence Scale [8]. This scale has been validated in multiple languages and has been used in various populations, including patients with chronic diseases [9]. The scale assesses medication adherence over the past seven days and includes questions about medication-taking behavior (Table 1). The score is calculated by assigning a score of one to each correct response to the eight items and a score of zero to each incorrect response. The scores are then summed to create a total score, with higher scores indicating greater medication adherence.

**Table 1:** Morisky Medication Adherence Scale

MMAS-8 Question	
1	Do you ever forget to take your medicine?
2	Are you careless at times about taking your medicine?
3	When you feel better, do you sometimes stop taking your medicine?
4	Sometimes if you feel worse when you take the medicine, do you stop taking it?
5	Do you ever change the dose of your medicine without asking your doctor?
6	Do you ever stop taking your medicine because you feel better?
7	Do you ever stop taking your medicine because you feel worse?
8	Do you ever have problems remembering to take your medicine?

Data analysis

The data collected from the survey were analyzed using the IBM Corp. Released 2021. IBM SPSS Statistics for Windows, Version 28.0. Armonk, NY: IBM Corp. Descriptive statistics were used to summarize the socio-demographic characteristics of the participants, their chronic disease diagnosis, and their medication adherence. In addition, ANOVA and t-tests were used to compare the medication adherence score among different categories such as age, gender, education level, marital status, employment status, and the presence of comorbidities. The significance level was set at p < 0.05 for all statistical tests.

Ethical consideration

This study followed the ethical principles outlined in the Declaration of Helsinki and relevant Kingdom of Saudi Arabia regulations. All participants provided informed consent and were given a clear explanation of the study's purpose. Participation was voluntary, and the survey did not collect any personally identifiable information to maintain the confidentiality and security of participants' data. All data collected were kept strictly confidential and securely stored, only accessible by the research team for the purpose of the study.

## Results

Demographic and clinical characteristics

A total of 400 participants were recruited for this study. Table 2 summarizes the demographic and clinical characteristics of the study population. The majority of the participants were female (62.5%), and the mean age was 46.2 years. Hypertension (48.8%) and diabetes (37.3%) were the most common chronic diseases reported.

**Table 2:** Demographic and clinical characteristics of the study population Income levels were collected in Saudi Arabian Riyals (SAR) and converted to United States Dollars (USD) using an exchange rate of 1 USD = 3.75 SAR for the purpose of reporting in this table.

	Characteristic	Number (%)
Gender	Male	150 (37.5)
	Female	250 (62.5)
Age (years)	18-29	68 (17.0)
	30-39	116 (29.0)
	40-49	95 (23.8)
	50-59	85 (21.3)
	≥60	36 (9.0)
Education	High school	160 (40.0)
	Bachelor’s degree	140 (35.0)
	Master’s degree or PhD	100 (25.0)
Income	Less than 2,666 USD	130 (32.5)
	2,666-5,333 USD	160 (40.0)
	Greater than 5,333 USD	110 (27.5)
Comorbidities	1	110 (27.5)
	2	170 (42.5)
	≥3	120 (30.0)
Chronic Disease	Hypertension	195 (48.8)
	Diabetes	149 (37.3)
	Asthma	51 (12.8)
	Other	5 (1.3)

Medication adherence score

**Figure 1:**
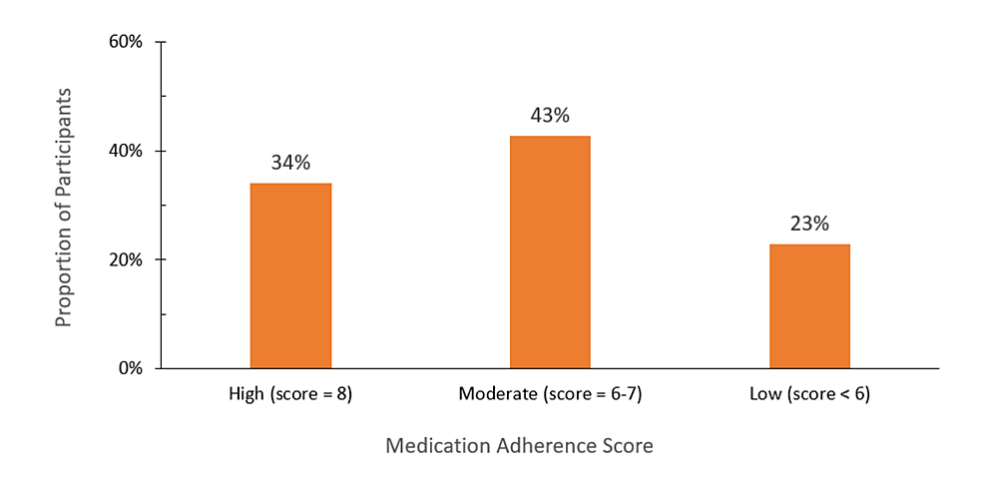
Medication adherence score distribution

Figure 1 shows the distribution of medication adherence scores. Of the 400 participants, 34.3% had high adherence (score of eight), 42.8% had moderate adherence (score of 6-7), and 22.9% had low adherence (score less than six). These categories were based on established cutoff scores for the scale.

Socio-demographic factors associated with medication adherence

The socio-demographic factors that were significantly associated with medication adherence were age, gender, and education level. Older age was positively associated with medication adherence (p = 0.01), indicating that older patients were more likely to adhere to their medications. Female patients were also more likely to adhere to their medications compared to male patients (p = 0.03). Patients with higher levels of education were also more likely to adhere to their medications (p = 0.04) (Table 3).

**Table 3:** Bivariate analysis results for demographic and clinical characteristics

	Characteristic	Medication Adherence Score	P value
Age (years)	18-29	3.8	0.01
	30-39	4.8	
	40-49	4.7	
	50-59	7.1	
	≥60	6.7	
Gender	Male	4.1	0.03
	Female	6.8	
Education	High school	3.8	0.04
	Bachelor’s degree	4.9	
	Master’s degree or PhD	5.2	
Income	< 10,000 SAR	6.8	0.25
	10,000-20,000 SAR	4.8	
	> 20,000 SAR	5.9	
Comorbidities	1	5.2	0.34
	2	5.8	
	≥3	6.4	
Chronic Disease	Hypertension	5.9	0.47
	Diabetes	6.4	0.25
	Asthma	4.9	0.34
	Other	5.8	0.85

Medication-related factors associated with medication adherence

Medication-related factors that were significantly associated with medication adherence included the number of medications prescribed, medication complexity, and medication cost. Patients who were prescribed more medications were less likely to adhere to their medications (p = 0.04). Patients who perceived their medication regimen as complex were also less likely to adhere to their medications (p = 0.03). Patients who reported that the cost of their medications was a financial burden were also less likely to adhere to their medications (p = 0.01) (Table 4).

**Table 4:** Bivariate analysis results for medication-related factors

Characteristics		Medication Adherence Score Mean	p-value
Number of Medications	<3	7.2	0.04
	3-5	4.5	
	>5	3.2	
Frequency of Medication	Once daily	6.8	0.03
	Twice daily	7.1	
	Three times daily	4.8	
	Four times daily	5.2	

## Discussion

The present study aimed to investigate factors related to medication adherence in patients with chronic diseases. The finding that medication adherence was significantly associated with age is consistent with some previous studies that have shown that older patients are more likely to adhere to their medications [10]. However, this finding may be different in different populations. One possible explanation is that different populations may have different attitudes and beliefs towards medication adherence, and these may vary depending on cultural, socioeconomic, and healthcare system factors.

The finding that medication adherence was significantly associated with gender is consistent with previous studies that have shown that female patients are more likely to adhere to their medications [11]. The reason for this gender discrepancy is unclear. It is possible that differences in health consciousness, health literacy, or cultural and societal norms may play a role. However, more research is needed to confirm these hypotheses and determine the best ways to improve medication adherence rates among both men and women with chronic diseases.

The finding that medication adherence was not significantly associated with the presence of comorbidities is inconsistent with previous studies that have shown that patients with multiple chronic diseases are more likely to be non-adherent to their medications [12]. One possible explanation is that our study sample had a higher education level. Another possible explanation is that the specific chronic diseases included in the study may not have been associated with medication non-adherence. The study may not have included a large enough sample of patients with certain comorbidities to detect a significant association with medication adherence.

The finding that medication adherence was significantly associated with educational level is consistent with previous studies that have shown that patients with higher educational levels are more likely to adhere to their medications [13]. Patients with higher educational levels may have a better understanding of the importance of medication adherence and the consequences of non-adherence. Therefore, healthcare providers should consider tailoring patient education and communication strategies based on the patient's educational level.

The finding that medication adherence was not significantly associated with income is inconsistent with previous studies that have shown that patients with higher incomes are more likely to adhere to their medications [14]. However, the present study did not collect data on the patient's health insurance status or out-of-pocket medication costs, which may have confounded the association between medication adherence and income.

In addition to patient-related factors, healthcare providers should also focus on healthcare system-related factors that affect medication adherence [15-18]. For example, the availability and affordability of medications, the accessibility of healthcare facilities, and the quality of healthcare services. In Saudi Arabia, the government provides universal healthcare coverage to all citizens, and medications for chronic diseases are available for free or at a low cost in government hospitals and clinics [19]. However, this study highlights the need for further improvement in the accessibility and quality of healthcare services to enhance medication adherence among patients with chronic diseases.

Medication adherence is critical for achieving treatment success in chronic diseases, but low adherence is a widespread issue globally. Non-adherence can lead to more complications and, in severe cases, even death. In Saudi Arabia, there is limited data available on medication adherence among chronic disease patients, and more information is needed to understand this issue better. Studies were conducted in Saudi Arabia using the Adherence to Refills and Medication Scale to identify adherence levels among chronic disease patients [20,21]. In these studies, the majority of patients (91.4%-96.62%) were non-adherent to their medications. In our study conducted in Jeddah, Saudi Arabia, 22.9% had lower adherence to medications. The difference in adherence levels observed in our study compared to previous studies conducted in Saudi Arabia may be due to several factors, such as differences in the demographic characteristics of the study populations, including age, gender, and disease severity, which could have influenced medication adherence. Additionally, differences in study design, such as recruitment methods, may have contributed to the variability in the results.

The findings of this study have important implications for healthcare providers and policymakers. First, the identification of socio-demographic and medication-related factors associated with medication adherence can inform targeted interventions to improve adherence among patients. For instance, interventions aimed at improving medication education among patients with low education levels or those with complex medication regimens may lead to better medication adherence. Additionally, interventions aimed at reducing medication costs for patients could improve medication adherence among those who perceive medication costs as a financial burden. Second, healthcare providers should consider older age, female gender, and higher education levels as potential predictors of better medication adherence when providing care for their patients. Overall, improving medication adherence can lead to better health outcomes, reduced healthcare costs, and improved quality of life for patients, highlighting the importance of addressing the factors identified in this study.

It is important to acknowledge that this study has several limitations that need to be considered. First, the online nature of the survey may have introduced bias in the sample selection. Convenience sampling was utilized in this study, which could have excluded certain subgroups of the population that do not have access to or are not proficient in using social media platforms. This could limit the generalizability of the findings to a broader population. Additionally, there may be concerns regarding the accuracy of self-reported data in online surveys, as participants may be more likely to provide socially desirable responses or may not fully comprehend the questions. Moreover, the online survey format may have restricted the ability of researchers to follow up and clarify or probe for further information about the responses provided. Finally, the cross-sectional design of the study prevents causal inferences from being drawn and only allows for associations to be identified. These limitations should be considered when interpreting the results of the study.

## Conclusions

The study provides insight into medication adherence among patients with chronic diseases in Jeddah, Saudi Arabia. The findings indicate that the majority of patients have moderate medication adherence. Older age, female gender, and higher education levels were associated with better medication adherence. Additionally, medication-related factors such as the number of prescribed medications, medication complexity, and medication cost were found to be significant predictors of medication adherence. These findings suggest that interventions aimed at improving medication adherence among patients with chronic diseases should take into account both patient-specific and medication-related factors. Further research is needed to better understand the underlying factors contributing to medication non-adherence in this population and to develop effective interventions to improve medication adherence and, ultimately, improve health outcomes.

## References

[1] Yach D, Hawkes C, Gould CL, Hofman KJ (2004). The global burden of chronic diseases: overcoming impediments to prevention and control. JAMA.

[2] Memish ZA, Jaber S, Mokdad AH, AlMazroa MA, Murray CJ, Al Rabeeah AA (2014). Burden of disease, injuries, and risk factors in the Kingdom of Saudi Arabia, 1990-2010. Prev Chronic Dis.

[3] Kleinsinger F (2018). The unmet challenge of medication nonadherence. Perm J.

[4] Seng JJ, Tan JY, Yeam CT, Htay H, Foo WY (2020). Factors affecting medication adherence among pre-dialysis chronic kidney disease patients: a systematic review and meta-analysis of literature. Int Urol Nephrol.

[5] Piña IL, Di Palo KE, Brown MT (2021). Medication adherence: Importance, issues and policy: A policy statement from the American Heart Association. Prog Cardiovasc Dis.

[6] Prabahar K, Albalawi MA, Almani L, Alenizy S (2020). Assessment of medication adherence in patients with chronic diseases in Tabuk, Kingdom of Saudi Arabia. J Res Pharm Pract.

[7] Llorca CV, Cortés Castell E, Ribera Casado JM (2021). Factors associated with non-adherence to drugs in patients with chronic diseases who go to pharmacies in Spain. Int J Environ Res Public Health.

[8] Moon SJ, Lee WY, Hwang JS, Hong YP, Morisky DE (2017). Accuracy of a screening tool for medication adherence: A systematic review and meta-analysis of the Morisky Medication Adherence Scale-8. PLoS One.

[9] Awwad O, AlMuhaissen S, Al-Nashwan A, AbuRuz S (2022). Translation and validation of the Arabic version of the Morisky, Green and Levine (MGL) adherence scale. PLoS One.

[10] Burnier M, Polychronopoulou E, Wuerzner G (2020). Hypertension and drug adherence in the elderly. Front Cardiovasc Med.

[11] Chen SL, Lee WL, Liang T, Liao IC (2014). Factors associated with gender differences in medication adherence: a longitudinal study. J Adv Nurs.

[12] Chang SM, Lu IC, Chen YC, Hsuan CF, Lin YJ, Chuang HY (2021). Behavioral factors associated with medication nonadherence in patients with hypertension. Int J Environ Res Public Health.

[13] Alkatheri AM, Albekairy AM (2013). Does the patients' educational level and previous counseling affect their medication knowledge?. Ann Thorac Med.

[14] Osborn CY, Kripalani S, Goggins KM, Wallston KA (2017). Financial strain is associated with medication nonadherence and worse self-rated health among cardiovascular patients. J Health Care Poor Underserved.

[15] Yoon S, Kwan YH, Yap WL (2023). Factors influencing medication adherence in multi-ethnic Asian patients with chronic diseases in Singapore: A qualitative study. Front Pharmacol.

[16] Kvarnström K, Westerholm A, Airaksinen M, Liira H (2021). Factors contributing to medication adherence in patients with a chronic condition: A scoping review of qualitative research. Pharmaceutics.

[17] Nduaguba SO, Soremekun RO, Olugbake OA, Barner JC (2017). The relationship between patient-related factors and medication adherence among Nigerian patients taking highly active anti-retroviral therapy. Afr Health Sci.

[18] Mondesir FL, Levitan EB, Malla G, Mukerji R, Carson AP, Safford MM, Turan JM (2019). Patient perspectives on factors influencing medication adherence among people with coronary heart disease (CHD) and CHD risk factors. Patient Prefer Adherence.

[19] Al-Hanawi MK, Alsharqi O, Almazrou S, Vaidya K (2018). Healthcare finance in the Kingdom of Saudi Arabia: A qualitative study of householders' attitudes. Appl Health Econ Health Policy.

[20] Kurdi S, Albannay R, Alsinan Z, Islam A (2021). Evaluation of medication adherence among patients with chronic diseases in Saudi Arabia. Int J Clin Pract.

[21] Alosaimi K, Alwafi H, Alhindi Y, Falemban A, Alshanberi A, Ayoub N, Alsanosi S (2022). Medication adherence among patients with chronic diseases in Saudi Arabia. Int J Environ Res Public Health.

